# An optimized approach for processing of frozen lung and lavage samples for microbiome studies

**DOI:** 10.1371/journal.pone.0265891

**Published:** 2022-04-05

**Authors:** Rosana Wiscovitch-Russo, Harinder Singh, Lauren M. Oldfield, Alexey V. Fedulov, Norberto Gonzalez-Juarbe

**Affiliations:** 1 Department of Infectious Diseases and Genomic Medicine, J. Craig Venter Institute, Rockville, Maryland, United States of America; 2 Department of Surgery, Division of Surgical Research, Rhode Island Hospital, Alpert Medical School of Brown University, Providence, Rhode Island, United States of America; Washington State University - Spokane, UNITED STATES

## Abstract

The respiratory tract has a resident microbiome with low biomass and limited diversity. This results in difficulties with sample preparation for sequencing due to uneven bacteria-to-host DNA ratio, especially for small tissue samples such as mouse lungs. We compared effectiveness of current procedures used for DNA extraction in microbiome studies. Bronchoalveolar lavage fluid (BALF) and lung tissue samples were collected to test different forms of sample pre-treatment and extraction methods to increase bacterial DNA yield and optimize library preparation. DNA extraction using a pre-treatment method of mechanical lysis (lung tissue) and one-step centrifugation (BALF) increased DNA yield and bacterial content of samples. In contrast, a significant increase of environmental contamination was detected after phenol chloroform isoamyl alcohol (PCI) extraction and nested PCR. While PCI has been a standard procedure used in microbiome studies, our data suggests that it is not efficient for DNA extraction of frozen low biomass samples. Finally, a DNA Enrichment kit was tested and found to improve the 16S copy number of lung tissue with a minor shift in microbial composition. Overall, we present a standardized method to provide high yielding DNA and improve sequencing coverage of low microbial biomass frozen samples with minimal contamination.

## 1. Introduction

The lower respiratory tract (LRT) was once thought to be a sterile environment [1–3]. Initial studies used culture-based methods, microscopy, and biochemical assays to isolate and identify microorganisms from the lung environment. However, the microbes isolated were mostly associated with pathogenic infections (e.g., *S*. *pneumoniae*, *H*. *influenzae*, *P*. *aeruginosa*, and *S*. *aureus* among others) or contamination during sample collection [1–3]. Recent developments in culture-independent methods rely on 16S ribosomal RNA (16S rRNA) and next-generation sequencing (NGS) aimed towards identification of viable but non-culturable bacteria [4]. The Human Microbiome Project (HMP) mainly used 16S rRNA based sequencing to study the oral, nasal, vaginal, gut, and skin microbial flora and improved the understanding of host-microbe interactions [5, 6]. While a better understanding of human microbial communities of multiple host sites is currently available, the microbial composition of lung flora and how the environment alters such is still to be elucidated.

Pioneering studies of the human lung microbiome used diverse methods for sample collection and processing (S1 Table). Due to tissue accessibility and ethical concerns, most human studies assessed the microbiome of the upper respiratory tract (URT) by collecting swab samples from the nasopharynx or pharynx [7, 8]. Although they are topologically distinct environments, recent studies have shown that URT is a close representation of the LRT housing similar microbiota but in distinct logarithmic quantities [9, 10]. It has been shown that the human lung has a resident microbiome of bacteria, fungi and viruses derived from nasopharyngeal commensals [11]. Importantly, compared to other niches (e.g., gut), the lung microbiome is limited in diversity and has a low microbial biomass (estimated 2.2 x 10^3^ bacterial genomes per cm^2^ of lung tissue), leading to difficulties in its study [11, 12]. Most human studies also present additional variables such as pre-existing health conditions (e.g. obesity, smoking, antibiotics, asthma or cancer), the difference in age groups and geographical locations which can influence the baseline microbiome of an individual [13, 14]. Of note, the host environment (rural vs. urban) and lifestyle (diet, smoking, etc.) highly influences the lung microbiome [8]. These intriguing effects of lung microbiome interactions with environment produce a lot of interest and are being actively studied. However, lack of consistent sample isolation and preparation procedures hampers our ability to compare studies [15, 16]. Animal models serve as strong tools to dissect microbiome changes specific to environmental exposures and the interaction between host and microbes in a controlled manner.

In addition to limited microbial diversity, low microbial biomass may reduce the detection of bacteria in mouse lung samples using 16S amplicon-based sequencing [17]. Most published studies using mouse models have shown some variability in lung microbiome profiles due to the use of different methods for sample processing, gene amplification and bioinformatic analysis [18–21]. Therefore, variability of normal lung microbiome of barrier laboratory mice includes not only the effects of strain, housing conditions and vendor [22, 23], but full contribution of sample pre-treatment is poorly studied [24]. It has been suggested that Phenol Chloroform Isoamyl alcohol (PCI) method is suitable for the low-biomass lung tissue investigation [25], while others have pointed out it leads to artefactual results in low-biomass specimens and is the most labor-intensive [26]. These two reports published last year emphasize the lack of cohesion in the matter. Here we tested different forms of processing for low-microbial-biomass frozen lung samples and bronchoalveolar lavage fluid (BALF) aiming to increase DNA yield and improve library preparation towards an optimized standardized method for sequencing of lung microbiomes.

## 2. Methods

### 2.1 Mice and sample collection

Six to nine -week-old female BALB/c mice were purchased from Jackson Laboratory (Bar Harbor, ME) or Charles River Laboratory (Wilmington, MA). Experiment was conducted in accordance with the Institutional Animal Care and Use Committee approved by the Brown University (Rhode Island Hospital IACUC #504718). Mice were housed in barrier conditions. Harvesting of lung tissue and/or bronchoalveolar lavage was performed under sterile conditions as described in [27, 28]. Briefly, mice were euthanized by intraperitoneal injection of Fatal-Plus, chest wall was rinsed with alcohol, the trachea was accessed via a cutaneous cut, soft tissues were separated blunt and a sterile angiocath catheter made of fluorinated ethylene propylene was inserted into trachea. Lavage was performed with sterile phosphate buffered saline (PBS) without Ca or Mg (Lonza), five repeat instillations of 0.5 mL. After lavage the chest wall was opened and the lungs were excised, washed in PBS and frozen.

### 2.2 Decontamination of workstation for sample processing and DNA extraction

Isolation of low microbial biomass from murine samples was done in a Class II Biosafety cabinet treated with Eliminase (DeconLabs, Cat# 1101) and 70% ethanol then exposed to UV light for 20–30 minutes to ensure sterile conditions. Additional suggestions for controlling contamination during sample processing:

#### 2.2.1 Frequently change gloves

During the procedure it is recommended to change gloves frequently. Change gloves after disinfecting the workstation and when solution spills on to gloves.

#### 2.2.2 Include controls during sample extraction

Include a water extraction (negative control) for each batch of samples processed to trace contamination back to the source when processing through the bioinformatic pipeline (Method 2.7). Extraction negative and cell mock community extraction (positive control) should be included in amplification and sequencing of all samples. ZymoBIOMIC Standards (Cat# D6300 and Cat# D6310) were used as extraction positive controls.

#### 2.2.3 Keep tube caps closed when not in use

Maintain all sample tubes closed at all times. Only open one tube at a time to discard or dispense the solution. Maintain distance between tubes while processing on the rack.

### 2.3 Phenol chloroform isoamyl alcohol DNA extraction

To pellet cells present in BALF samples, 1000 μL of the total sample volume was centrifuged (8,000xg for 15 minutes at 4°C), then resuspended in 500 μL of lysis buffer (20 mM Tris-Cl, pH 8.0, 2 mM EDTA, 1.2% Triton X-100). Separately, lung samples (left lobe) were submerged in 500 μL of lysis buffer. All samples, both tissue and BALF, were then incubated at 75°C for 10 minutes. After cooling at room temperature, samples were transferred into a Matrix B Lysing tube and further lysed in a BioSpec Mini-Beadbeater for 45 seconds. The obtained lysate was then treated with 60 μL of lysozyme (Sigma Aldrich, Cat# L6876-10G) and 5 μL of Linker RNase A (ThermoFisher, Cat# 12091039) and incubated at 37°C for 60 minutes. Subsequently, 100 μL 10% Sodium Dodecyl Sulfate (SDS) and 42 μL Proteinase K (QIAGEN, Cat# 19131) were added and incubated overnight at 55°C. After completing lysis protocol, we proceeded to isolate the DNA. Samples were treated twice with equal volumes of phenol chloroform isoamyl alcohol and cleaned through ethanol precipitation [29]. DNA was eluted in 50 μL of Tris-EDTA (TE) buffer and DNA concentration was measured using a Qubit 1X dsDNA HS Assay (ThermoFisher, Cat# Q33231).

### 2.4 Increasing extraction efficiency through sample pre-treatments

**Fig 1:**
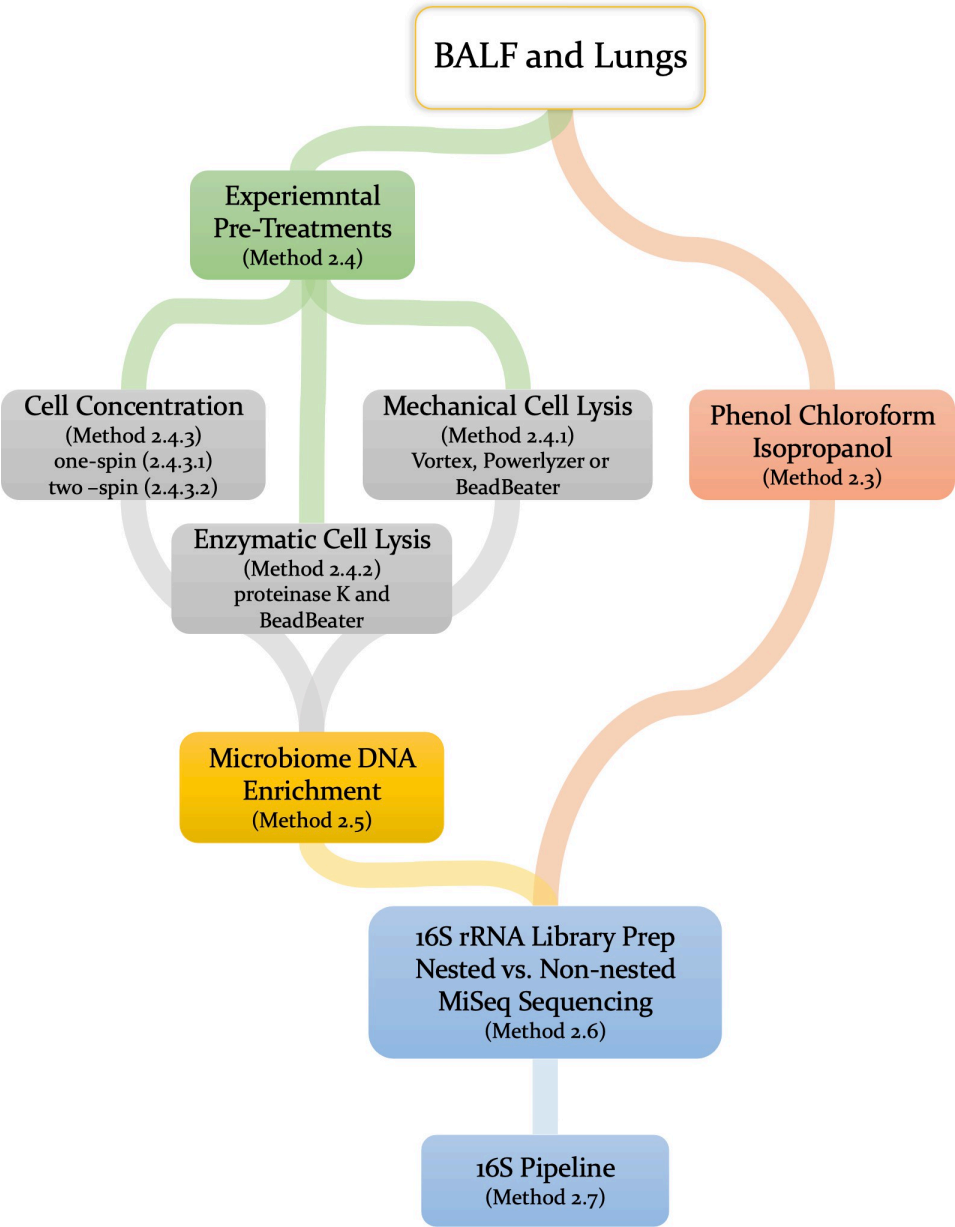
Workflow for processing BALF and lung samples for microbiome data. Initially we compared two DNA extraction methods (experimental kit-based and PCI) and three forms of sample pre-treatment method. A small subset of samples was used to compare the efficiency of Microbial Enrichment kit. The V4 16S region was selected for preparing two nested (25x25 and 35x25 PCR cycles) and one non-nested (35 PCR cycles) libraries preparation methods.

To extract DNA from 500–1500 μL of BALF and lung tissue (left lobe) samples were processed using the DNeasy PowerSoil Pro Kit (QIAGEN, Cat# 47016). To examine extraction efficiency, the step 2 of the of PowerSoil protocol was modified (Fig 1). Briefly:

#### 2.4.1 Mechanical cell lysis

First, 800 μL of CD1 lysis buffer provided by the kit was added to the collected BALF and lung tissue in PowerBead Pro tubes. Then, as described in the DNeasy PowerSoil Pro Kit protocol, disruption of the cell membrane was done by physical lysis via a Vortex-Genie 2 with a 24 sample adaptor at maximum speed for 15 minutes. In addition to that mentioned in the protocol, we treated the samples either by using a BioSpec Mini-Beadbeater at maximum speed for 45 seconds or using the QIAGEN PowerLyzer at 3000 rpm for 30 seconds.

#### 2.4.2 Enzymatic cell lysis

Further disruption of the cell membrane was done through enzymatic cell lysis using Proteinase K. Here, lung tissue samples were lysed in the provided PowerBead Pro tubes with 200 μL of TE buffer and 100 μL 10% SDS using the BioSpec Mini-Beadbeater at max speed for 45 seconds. Then, 40 μL of proteinase K was added and incubated overnight at 55°C.

#### 2.4.3 Cell concentration

An additional set of BALF samples were processed to concentrate biological material and/or to reduce host cell presence. To do the latter we used either one or two-step centrifugation processes:

*2*.*4*.*3*.*1 One-spin*. One-step centrifugation: samples were centrifuged once at 5,000xg for 5 minutes at 4°C to pellet all the cells (mammalian and bacterial).

*2*.*4*.*3*.*2 Two-spin*. Two-step centrifugation, (i) samples were centrifuged first at 300xg for 10 minutes at 4°C to pellet host cells, (ii) then supernatant was transferred into a new 1.5mL Eppendorf and centrifuged at 5,000xg for 5 minutes at 4°C to collect remaining bacterial cells.

The supernatant was collected and stored at -80°C for future use. The final pellets were resuspended in 800 μL of CD1 buffer and transferred to the provided PowerBead Pro tubes. The samples were then mechanically lysed using BioSpec Mini-Beadbeater at max speed for 45 seconds as described in 2.4.1.

For all lysed samples (see above), steps 3–17 of the PowerSoil protocol were carried out without further modifications except for the final step when DNA was eluted in 50 μL of C6 buffer (10 mM Tris) instead of 100 μL. DNA concentration was measured through Qubit 1X dsDNA HS Assay and Nanodrop ND-1000 Spectrophotometer.

### 2.5 Microbiome DNA enrichment kit

Purified gDNA extracted using the PowerSoil Kit (Methods 2.4.1 and 2.4.3.1) was split into two aliquots for the purpose of comparing the efficiency before and after treatment with the NEBNext Microbiome DNA Enrichment Kit (Cat# E2612S). This kit aims to increase bacterial DNA by selectively targeting and reducing host methylated DNA [30]. NEBNext Microbiome DNA Enrichment Kit was carried out according to manufacturer protocol. Briefly, 25 μL of BALF and 3–5 μL of lung gDNA was used during the procedure. Due to the high DNA concentration of lung tissue, aliquoted lung gDNA sample was diluted 1:10 in qPCR grade water, then 1 μL was used to match the DNA to bead ratio optimized in the manufacturer’s protocol. Target DNA was purified through ethanol precipitation and eluted in 25 μL of 1X TE buffer as the final step of the manufacturer’s protocol.

### 2.6 V4 16S amplification and sequencing

Purified gDNA from PCI method, PowerSoil Pro and Microbiome Enrichment Kits was used to amplify the hypervariable V4 region of the 16S rRNA gene comparing nested and non-nested PCR library preparation methods. Taxonomic profiling of the mice lung microbiome was based on the V4 16S rRNA hypervariable region because of its taxonomic resolution and accurate assignment [31–33]. The primers used were 515-533F forward (GTGCCAGCMGCCGCGGTAA) and 806-787R reverse (GGACTACHVGGGTWTCTAAT) [53]. The nested libraries were first prepared by amplifying the target region using non-barcoded primers at 25 or 35 cycles, then the second round of amplification used the barcoded primers at 25 cycles. The nested and non-nested libraries used the Illumina MiSeq adapter- and barcode-ligated dual-index primers [31]. PCR was performed using ThermoFisher Platinum Taq DNA Polymerase (Life Technologies, Cat# 10966–026); 94°C for 5 minutes, 94°C for 30 seconds, 55°C for 30 seconds, 72°C for 30 seconds for 25–35 cycles, 72°C for 7 minutes. Libraries were purified using SPRIselect beads (Beckman, Cat# B23318) for size selection of 300 bp fragments then quantified using ThermoFisher SYBRGold (Cat# S11494) in a TECAN microplate reader. Afterwards libraries were normalized, pooled (3.5 pM), and sequenced on Illumina MiSeq system using a V2 500 (2x250 bp) cycles chemistry kit supplemented with 20% PhiX.

### 2.7 Bioinformatic pipeline

We filtered poor quality reads (>Q30), trimmed primers and removed reads of <220 bp prior to analysis. Assigned taxonomy using in-house pipeline supported by Mothur [34] and Uparse [35]. Operational taxonomic units (OTUs) were based on SILVA 16S rRNA database version 123 at 97% sequence similarity [36]. We also used R statistical environment to generate plots using PhyloSeq package [37] and ggplot 2 packages to assess the microbial composition [38].

## 3. Results

BALF and lung samples underwent differential extractions, pre-treatments, and library preparation prior to 16S sequencing (Fig 1, workflow). A total of 50 samples were processed using two forms of DNA isolation methods: an experimental DNA isolation method (Method 2.4) that has a relatively short workflow, or the traditional and more laborious phenol chloroform isoamyl (PCI) DNA extraction method (Method 2.3). To increase DNA yield from these samples, we examined different experimental pre-treatment methods by modifying steps from an extraction kit protocol to test the lysis efficacy of various bead beating equipment (Method version 2.4.1). We also tested additional disruption of the cell membrane by enzymatic lysis (Method version 2.4.2) and concentrating the cells prior to mechanical lysis (Method version 2.4.3). Moreover, in separate experiments we tested the NEBNext Microbiome DNA Enrichment Kit aiming to improve bacterial-to-host DNA ratio and enhance primer specificity during library preparation (Method 2.5). For samples with low microbial biomass, we amplified the V4 16S region under nested and non-nested PCR conditions (Method 2.6). Finally, all samples underwent 16S sequencing and the microbiome was profiled using an in-house bioinformatics pipeline and R environment (Method 2.7).

### 3.1 Nested-PCR promotes DNA contamination from environmental organisms

Negative controls (see Method 2.2.2) were included in batch extractions. This is done as part of our bioinformatic pipeline, where negative controls are examined first to identify and remove contamination. The latter assures the observation of the true microbial community in a sample and aims to exclude microbial contamination. We tested 3 PCR conditions for all the samples: PCR-1 (one round of 35 cycles), PCR-2 (round 1 of 35 cycles, round 2 of 25 cycles), PCR-3 (round 1 of 25 cycles, round 2 of 25 cycles). Negative control samples showed a significant (p < 0.01) increase of reads upon nested (PCR-2) when compared to the non-nested PCR (PCR-1) (S1A Fig). To further analyze the biomass of 16S RNA copy number, we plotted the read count for each genus after nested or non-nested PCR conditions. The data showed that while an increase in reads was obtained, there was a higher amount of DNA contamination potentially from the kit’s reagents (e.g. *Pseudomonas* and *Shewanella* sp.) or trace amount of cross contamination from loci other than lungs (e.g. *Lactobacillus* sp. and *Enterobacteriaceae*) (S1B Fig) [39, 40]. We suggest this should be taken into consideration when working with samples that are naturally low-biomass and are easily susceptible to contamination. Hence, we only used the non-nested approach to evaluate pre-treatments and extraction methods, which we detail next.

### 3.2 Evaluating the effects of physical or chemical treatments to improve DNA content in frozen low microbial biomass samples

Pre-treatment methods that yield a higher DNA and bacterial content improves the chances for better representation of the lung microbial community [41]. We tested the effectiveness of two different mechanical and chemical DNA extraction methods.

#### 3.2.1 BALF samples

**Fig 2:**
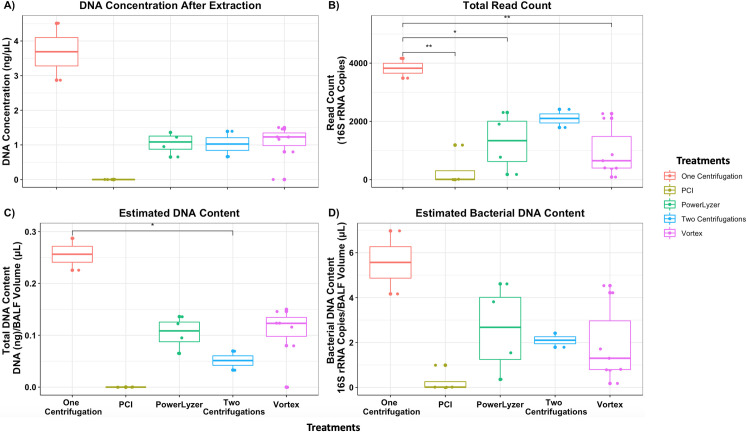
DNA content and estimated biomass of BALF samples. **A)** DNA concentration (ng/μL) of BALF samples, **B)** total read count is estimated based on the 16S rRNA gene copy number after quality filtering of the data, **C)** estimated DNA content dividing total DNA extracted by volume of the sample processed, and **D)** estimated bacterial DNA content dividing read count by volume of the sample processed. Significance (p-value) between groups is indicated by stars (* <0.05 and ** < 0.01). Based on overall data, most effective pre-treatment method for BALF samples was a one Method centrifugation followed by mechanical cell lysis. Two Method centrifugation was less effective.

**Fig 3:**
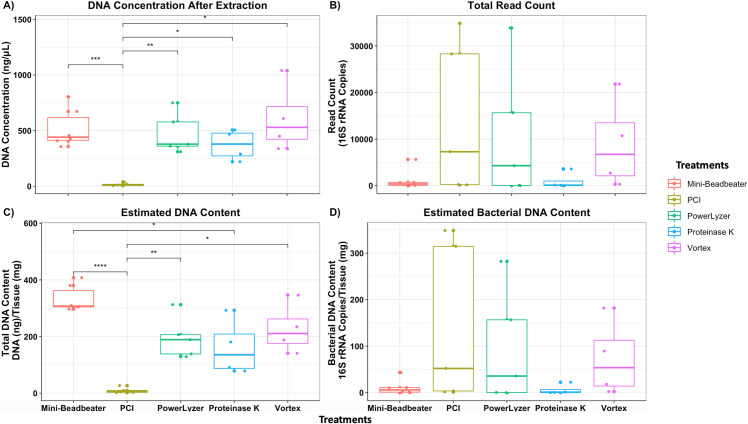
DNA content and estimated biomass of lung tissue samples. **A)** DNA concentration (ng/μL) of lung tissue samples, **B)** total read count is estimated based on the 16S rRNA gene copy number after quality filtering of the data, **C)** estimated DNA content dividing total DNA extracted by weight of the sample processed, and **D)** estimated bacterial DNA content dividing read count by weight of the sample processed. Significance (p-value) between groups is indicated by stars (* <0.05, ** < 0.01, *** < 0.001, and **** < 0.0001). Overall, most mechanically lysed tissue samples resulted in high DNA yield with exception of the PCI treated samples.

BALF samples had the lowest DNA concentration (Fig 2A) and read count (Fig 2B) compared to lung tissue samples (Fig 3A and 3B). The total DNA content (total DNA extracted by the volume of the sample processed) was the lowest for PCI extracted samples and higher for two-step centrifugation followed by bead-based mechanical cell lysis (Fig 2C). Similar outcomes were observed for estimated bacterial content per volume (μL) of samples processed using different forms of pre-treatments and methods (Fig 2D). Pre-treatment by one-step centrifugation resulted in the highest DNA concentration (average of 3.7 ng/μL) and bacterial content (average of 3825 reads) of the BALF samples. Overall, PCI was the least effective method for extracting DNA from BALF samples, the mean bacterial DNA content was estimated at 301 reads associated to contamination potentially from the reagents (S2 Fig). In contrast, the pre-treatment of BALF samples with one-step centrifugation followed by bead-based mechanical lysis (Method 2.4.3.1) increased the DNA concentration (Fig 2A) and significantly increased 16S read count when compared to other extraction methods (Fig 2B). Thus, one-step centrifugation followed by bead-based mechanical lysis of BALF samples was the more efficient and high yielding DNA extraction method and thus produced a higher 16S copy number library.

#### 3.2.2. Lung tissue samples

Lung tissue samples naturally had higher total DNA concentration because of the high amount of host DNA (Fig 3A). Furthermore, host DNA did not inhibit the specificity of the V4 16S primers (Fig 3B). The mean DNA concentration of the lung tissue sample per pre-treatment method was as follows: Vortex (609 ng/μL) > Mini-Beadbeater (520 ng/μL) > PowerLyzer (475 ng/μL) > Proteinase K (371 ng/μL) > PCI (15 ng/μL). While the mean 16S read count was as follows: PCI (14,176 reads) > PowerLyzer (10,781 reads) > Vortex (8,899 reads) > Mini-Beadbeater (1,193 reads) > Proteinase K (960 reads). Mechanical cell lysis of lung tissue using the Mini-Beadbeater resulted in improved total DNA content per weight (mg) (Fig 3C). However, PowerLyzer and Vortex-Genie 2 produced a higher estimated bacterial content per weight (mg) than the Mini-Beadbeater (Fig 3D), suggesting they are more efficient in extracting high yielding DNA and 16S copy number for lung tissue samples. PCI method resulted in a low DNA concentration of lung tissue samples (Fig 3A) and bacterial reads obtained from PCI were substantially associated with contamination potentially from the reagents used during extraction [28] (S2 Fig). We further validated the effectiveness of the commercial kit method on the positive controls (ZymoBIOMICS Mock Community), which effectively lysed representative bacterial species resulting in similar compositions as the one suggested by the ZymoBIOMICS 16S rRNA gene abundance (S3 Fig). Overall, the most effective extraction method for frozen lung tissue samples was mechanical cell lysis using bead-based equipment (i.e., PowerLyzer or Vortex-Genie 2).

### 3.3 Evaluating bacterial diversity of distinct sample types

**Fig 4:**
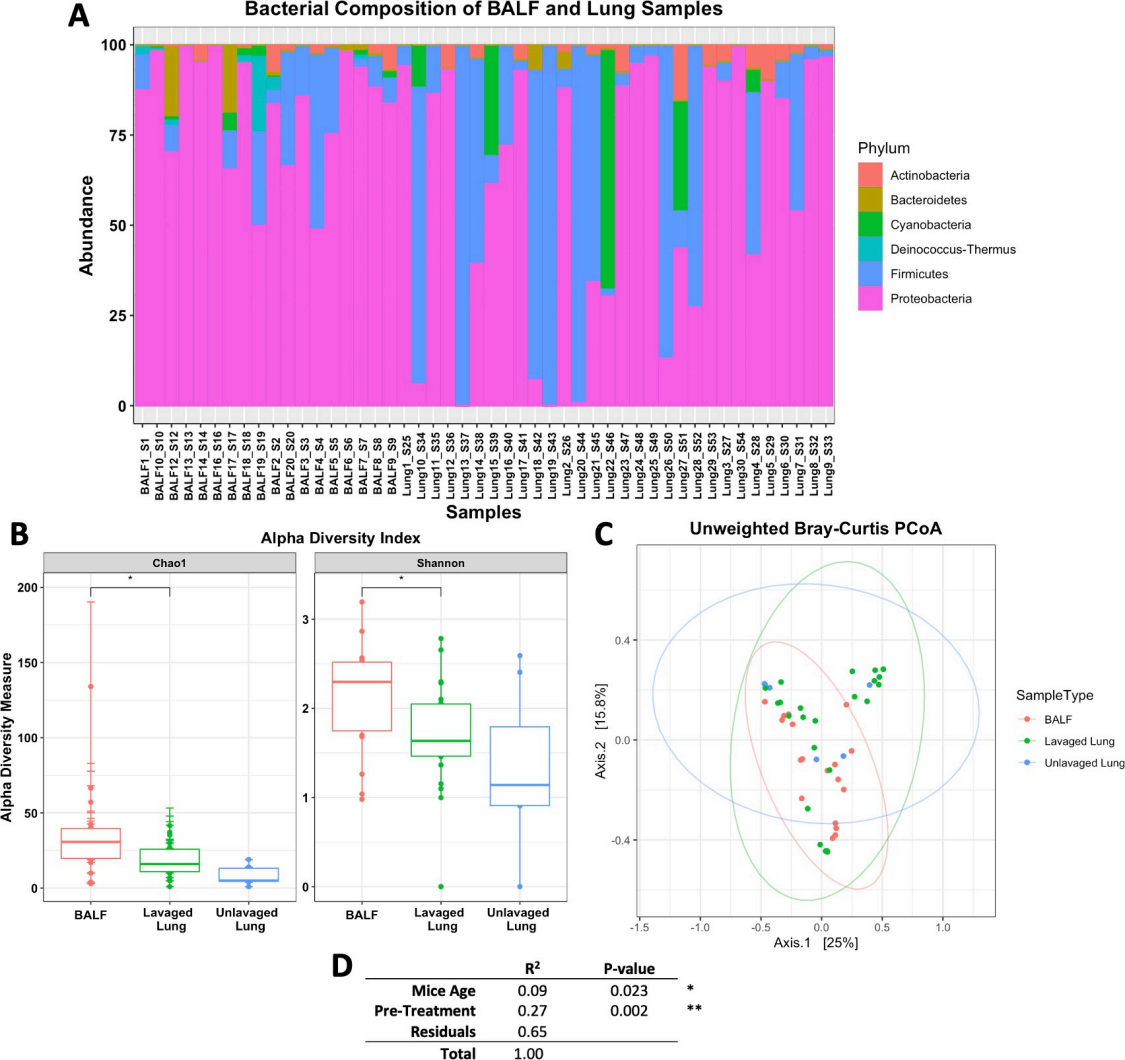
Diversity analysis of BALF and lung tissue samples. **A)** Bacterial composition of lung and BALF samples. Overall, samples had a higher abundance of Proteobacteria and Firmicutes bacteria. **B)** Alpha diversity measures shows that BALF samples have a higher species richness compared to both lavaged and unlavaged lung samples. Observed significant (p < 0.05) difference in species richness between BALF and lavaged lung samples. **C)** Beta diversity plot shows admixture of sample types. **D)** Statistical summary of ADONIS test shows the variance and significance when comparing mice age and extraction pre-treatment methods.

At phylum level, we observed that BALF and lung tissue samples had similar microbial profiles, even if each was processed using different forms of pre-treatment methods. Bacteroidetes, Firmicutes and Proteobacteria were abundant among all samples (Fig 4A). We used two types of lung tissue samples, unlavaged and lavaged. In addition, we compared these lung samples with BALF. According to alpha diversity measures, species richness is more abundant in the BALF than in the lavaged lung samples (p < 0.05) (Fig 4B). Surprisingly, lavaged lungs had only slightly lower diversity than unlavaged lung samples (Fig 4B). Overall, beta diversity plots demonstrated similar microbial composition in the three sample types (Fig 4C). Permutation test suggests that sample pre-treatment significantly (p-value 0.002) influenced the microbial communities (Fig 4D). Samples were sequenced on the same MiSeq lane, thus reducing bias introduced by the difference in sequencing depth. Overall, BALF samples showed the most efficient way to test the pulmonary microbiome.

### 3.4 Microbiome DNA enrichment kit improves recovery of microbial DNA from frozen lung tissue samples

**Fig 5:**
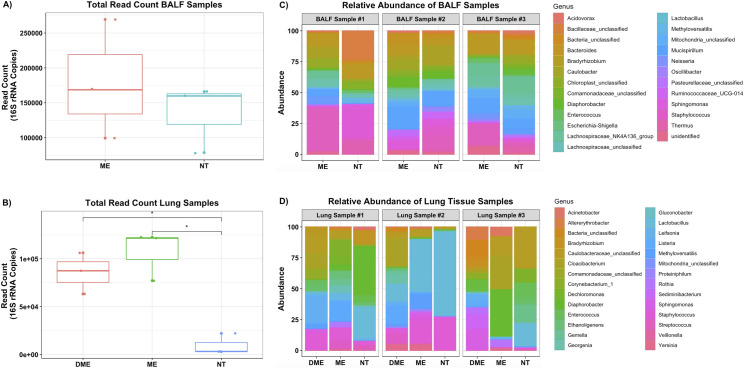
Comparing the efficiency before and after treatment with the NEBNext microbiome DNA Enrichment Kit. **A)** No significant improvements observed when comparing microbiome enriched (ME) and non-treated (NT) BALF samples total read count. **B)** However, significant improvements observed when comparing DME (1:10 diluted sample then treated with ME kit), ME, and NT lung samples total read count. Significance (p-value) between groups is indicated by stars (* <0.05 and ** < 0.01). Both **C)** BALF and **D)** lung samples showed difference in community composition (genus level) between NT and ME samples, particularly diluted lung samples treated with microbiome enrichment kit.

To improve 16S library prep of low microbial biomass samples, NEBNext Microbiome DNA Enrichment Kit was used to increase the bacterial to host DNA ratio. This kit depletes host DNA by targeting and reducing methylated host DNA from the sample [42]. Select samples were divided into two DNA aliquots to compare the effect of the NEBNext kit. No significant change of the 16S copy number was observed in the enriched BALF samples (Fig 5A). However, significant (p < 0.05) improvement was observed for the microbiome enriched lung tissue samples (Fig 5B). When comparing microbial composition, differences in bacterial profiles were observed at genus level between non-treated and NEBNext treated samples. These differences in genus level profiles were more prominent in the lung tissue than in the BALF, primarily when using diluted DNA (Fig 5C and 5D). The NEBNext treated BALF samples closely resembled the microbial composition of the untreated BALF samples at the genus level (Fig 5D). Bacteria identified in NEBNext treated tissue resembled the profile observed in BALF at phylum level (Figs 4A and S4C). Taken together, our data shows that the NEBNext increases bacterial DNA yields of lung tissue samples. However, slight differences in recovered microbial profiles were observed between NEBNext treated and non-treated samples.

## 4. Discussion

The purpose of this study was to develop an optimal method of sample homogenization, DNA extraction and library preparation for low microbial biomass samples. Culture-based methods estimate that murine lungs harbor about 10^3^–10^5^ colony forming units (CFU) per gram of lung tissue [43]. Having a low microbial density and at the same time a complex tissue structure, lung sample pre-treatment methods are necessary to effectively lyse all cell types and increase the DNA yield. The estimated DNA and bacterial content is used to determine effective pre-treatment methods for the samples. Here we aimed to define the most efficient method to ensure isolation of high yield DNA from low microbial biomass samples to effectively study the pulmonary microbiome.

We first tested two DNA extraction methods, an experimental DNA isolation method with a short workflow and the traditional PCI. Currently, PCI is the most common and cost-efficient method for DNA isolation used in microbiome studies. The method claims to deliver high yielding and quality DNA [25]. Here we tested PCI isolation method to examine the latter claim and compare to an extraction kit to establish a standard protocol for DNA extraction of low microbial biomass samples. Overall, PCI isolation method produced a lower DNA yield resulting in a poor representation of the pulmonary microbial communities after 16S sequencing. Of note, in the PCI method a high level of bacterial DNA was isolated from the contaminated reagents causing to suppress the 16S signal of the lung microbial community. Similar observations have been shown in studies targeting the placental or sputum microbiome, where reagent contamination had an effect on the microbial profiles [26, 44]. PCI method was also found not ideal for DNA isolation of low biomass samples due to multiple treatments and washing steps which resulted in DNA loss [45]. In addition, trace amounts of phenol may be carried over and potentially interfere with the downstream processing of the sample [46, 47]. In sum, PCI isolation method is not optimal for processing low biomass samples such as those targeting lung microbiome analysis.

Compared to the PCI method, the experimental method using a combination of bead-based lysis and an extraction kit, resulted in minimal sample loss and contamination. In contrast to other commercial extraction kits, QIAGEN PowerSoil Pro was used since it can be applied to a broad range of sample types (in addition to soil or stool samples) and customizable protocol. Our experimental approach that further customized and improved the kit’s ability to extract high quality DNA. Other extraction kits, like the Molzym Ultra-Deep Microbiome Prep, QIAamp DNA Microbiome and Zymo HostZERO microbial DNA kits (among others), have been used as an alternative to process samples containing a low amount of microbial biomass with variable results [42, 48]. These kits selectively lyse host cells and enzymatically (mostly using Dnase) deplete host DNA assuring high yielding bacterial DNA [42, 49]. However, the efficiency of such kits may be tied to the use of fresh samples. Freeze-thawing cycles compromise the integrity of bacterial cell membrane [50] and thus expose bacterial DNA can be degraded along with the host DNA in the enzymatic depletion step. The samples used in the presented study were previously frozen and the bacterial cell wall integrity has been compromised through freeze-thaw cycles, by using the pre-treatments and kit extraction method we were able to effectively isolate high yield DNA in a manner independent of membrane integrity. Thus, our experimental approach provides an ideal methodology for groups assessing microbial communities in samples that have undergone freezing and long-term storage. Taken together, the experimental DNA isolation method was found to provide a fast and reliable workflow that allows for streamlined sample processing, isolate high yielding DNA and is ideal for low biomass frozen samples.

Having a low microbial density, sample pre-treatment methods are necessary to increase DNA yield of mice lung samples. According to the overall yield (DNA concentration and read count), lung tissue samples were efficiently processed by mechanical lysis by using either PowerLyzer or Vortex-Genie 2 equipment. Overall, the PowerLyzer or Vortex-Genie 2 are equally effective at lysing tissue cells because of the unique motion and horizontal tube positioning of the equipment. DNA isolation from BALF samples was successful using one-step centrifugation followed by mechanical lysis of the cells. Previously published data had shown a two-step centrifugation process to initially collect heavy mammalian cells at a low speed, followed by a second centrifugation step at a higher speed to only collect bacterial cells had an impact on microbiome profiles of BALF [18]. In contrast, in our hands, one-step centrifugation of BALF was more efficient in increasing DNA yield, since both host cell-attached and free-living microbe DNA was isolated. Of note, many bacterial species (e.g. *Salmonella*, *Yersinia*, *Pseudomonas*, *Klebsiella*, *and Haemophilus* sp.) have been shown to strongly adhere to host cells [50]. Moreover, discarding host cells prior to isolating DNA for a microbiome study deprives of any information regarding intracellular microbes (e.g. *Chlamydia* and *Mycoplasma sp*.), which can be essential in lung research [51]. Furthermore, pelleting the cells does not affect the reaction volume or the downstream chemistry of the kit. We suggest a one-step centrifugation protocol as a more representative of the overall pulmonary microbial community.

For library preparation, we experimented with nested PCR at different cycle numbers and observed higher level of contamination. Similar to our findings, Drengenes *et al*. showed that during library preparation for human pulmonary samples, nested PCR increased contamination causing a decrease in 16S signal [52]. In our study, contamination was more evident in the negative controls, nested PCR more than doubled the amplification of DNA contamination compared to the non-nested negative controls. In fact, other studies (metagenomics and 16S-based analysis) have stressed the importance and use of negative and other extraction controls to distinguish the source of contamination in low microbial biomass samples [44, 53, 54]. Besides nested PCR, other library preparation methods are recommended to increase 16S coverage of low microbial biomass samples with minimal amplification of environmental contaminants. For instance, slightly increasing PCR cycle number (from 35 to 40 cycles) or doubling library preparation of a sample can improve the 16S copy number [18, 54]. Whichever method is decided, the use of positive and negative controls during DNA extraction and library preparation is key for tracking contamination to the source, which can be later removed using diverse bioinformatic tools.

Considering the excess amount of host nucleic acid after DNA extraction from lung tissue and BALF, we expected loss of specificity and reduced annealing of the16S rRNA primers during library preparation. We aimed to improve host-to-bacteria DNA ratio using an enrichment kit to determine if the representation of the pulmonary microbial community in our frozen samples could be further improved. A subset of the samples was processed using the NEBNext Microbiome DNA Enrichment kit to deplete mammalian host derived DNA. Initially, we attempted to measure the efficiency of the kit using a SYBR Green qPCR approach to assess the host-bacteria DNA ratio by targeting host glyceraldehyde 3-phosphate dehydrogenase (*Gapdh*) and 16S rRNA genes. The assay was unsuccessful due to the low sensitivity of 16S SYBR Green qPCR assay (S5 Fig) which has a detection limit estimated at 100 bacterial cells [55, 56]. The latter results suggest SYBR Green may not be sensitive enough to determine the quality of the isolated DNA. In contrast, the MiSeq sequencing data provided information on the 16S rRNA copy number between NEBNext treated and non-treated samples. Host depletion was found more successful on the lung samples due to the high amount of tissue and immune cell types [11, 12]. In BALF samples, the overall reduced presence of host cells did not alter recovered 16S copy numbers. Therefore, it is unnecessary to treat BALF samples or other low host DNA samples (like other lavages or swabs) with the NEBNext kit, reducing cost of sample processing [30–57]. When analyzing 16S data, we observed slight differences in community composition between NEBNext treated and non-treated lung tissue samples. Similar to our findings, other studies have shown changes in microbial profiles when treating human sino-nasal swab, saliva or blood samples with the enrichment kit [30–57]. The commercial kit, marketed as targeting and reducing host DNA, could potentially be skewing the microbiome data by removing methylated bacterial DNA in the process [58]. Additionally, further diluting the sample, resuspended NEBNext treated bacterial DNA in a larger volume compared to input volume of gDNA, could be further skewing the microbiome data. However, further investigation is required for these claims. Importantly, microbial composition of BALF and NEBNext treated lung tissue was similar, suggesting that BALF may be the most efficient way to assess the pulmonary microbiome.

Overall, our work suggests that the optimal processing of murine lung tissue for microbiome analysis is by mechanically lysis the sample using bead-based equipment (PowerLyzer or Vortex-Genie 2), extraction kit following manufacturer’s instructions post-lysis steps, and if possible, treating the samples with NEBNext Microbiome DNA Enrichment Kit. For BALF samples we observed increased DNA recovery by centrifugating the samples (once at 5,000xg) followed by mechanical lysis and extraction kit following manufacturer’s instructions post-lysis steps. For all sample types, the microbial profile was successfully represented with minimal contamination using a library preparation method with 35 PCR cycles (non-nested PCR).

## 5. Conclusion

We have developed a customized protocol to efficiently extract and 16S-sequence mice BALF and lung tissue samples to study the pulmonary microbiome. Alternately, the protocol can also be applied to other tissue and lavage samples with low microbial density. The protocol can be used to study the effects of the environment or disease state between host and microbes.

## Supporting information

S1 Table Brief description of cited article’s methodology and findings. (PDF)

S1 Fig Total read count (16S rRNA copy number) of extraction negative controls. **A)** Data shows that nested PCR more than doubles amplification of contamination. Significant (p < 0.01) difference observed when comparing nested (35x25) and non-nested PCR. **B)** Nested samples exponentially increased contamination in all negative control. Thus, nested PCR samples were discarded form the analysis and from further processing.
(PDF)

S2 Fig Total read count (16S rRNA copy number) from PCI extracted samples. Most samples had a high level of contamination potentially from water and reagents by detecting a higher abundance of *Pseudomonas* and *Shewanella* sp. overshadowing the lung microbiome signal in most samples.
(PDF)

S3 Fig Examine extraction efficiency of pre-treatment methods on ZymoBIOMICS microbial community standards. **A)** Mock Community Standard I (Cat# D6300) and **B)** Mock Community Standard II (Cat# D6310), all pre-treatment methods give a good representation of the microbial mock community as suggested by ZymoBIOMICS 16S theorical microbial composition.
(PDF)

S4 Fig Diversity analysis of treated and non-treated samples with the NEBNext Microbiome DNA Enrichment Kit. Abbreviation for non-treated (NT), microbiome enriched (ME) and diluted sample then treated with ME kit (DME). **A)** Alpha diversity measures shows that BALF samples, treated samples have a lower species diversity comparted to the non-treated samples. **B)** Alpha diversity measures shows that lung tissue samples, slight increase species richness was observed for treated lung samples. **C)** Bacterial composition of lung and BALF samples. Overall, samples had a higher abundance of Proteobacteria and Firmicutes bacteria.
(PDF)

S5 Fig Comparing 16S/GAPDH primers and controls for Sybr Green qPCR assay. Both 16S and GAPDH genes had a low primer specificity in the qPCR assay. For 16S qPCR assay, cutoff was set to 100 copies. GAPDH cutoff was set to 1000 copies.
(PDF)

S1 Data (XLSX)
