# Supplementary material for: An optimized approach for processing of frozen lung and lavage samples for microbiome studies
Source: PLoS One. 2022 Apr 5;17(4):e0265891. doi: 10.1371/journal.pone.0265891 (PMC8982836; doi:10.1371/journal.pone.0265891)
Supplement: S1 Table — (PDF) [file pone.0265891.s001.pdf]

Subjects	Population	Sample No	Age	Gender	Sample Type	Microbiome Profile	Citation
**Development**	Netherlands	60	1.5-24 months	Male and Female	Nasopharyngeal Aspirates	1.5 months ↑*Streptococcus, Moraxella, Staphylococcus, Corynebacterium* and *Dolosigranulum*<br>6 months ↑*Staphylococcus, Haemophilus, Moraxella, Corynebacterium* and *Dolosigranulum* spp.<br>12-24months ↑*Staphylococcus, Haemophilus,* and *Moraxella* spp.	**1**
	USA	7	26-45 years	Male and Female	Nasal and Oropharynx Swab	↑Firmicutes (Streptococcaceae, Lachnospiraceae, and unclassified Clostridia group), Proteobacteria, and Bacteroidetes	**2**
	Canada	260	18-96 years	Male and Female	Nasal and Oropharynx Swab	mid-aged adults (18-45 years) ↑*Streptococcus*, *Prevotella*, and *Veillonella*<br>elderly (>65 years) ↑*Streptococcus, Lactobacillus* and *Lactococcus*	**3**
**Lung Topology**	USA	6	24-65 years	Male and Female	Oral Wash, Pharyngeal Swabs and BAL	↑Streptococcaceae, Prevotellaceae, Veillonellaceae, Fusobacteriaceae, Neisseriaceae, Propionibacteriaceae, and Lachnospiraceae	**4**
	USA	28	-	Male and Female	Oral Wash, Nasal Swabs, BAL and Gastric Aspirate	↑*Prevotella*, *Streptococcus*, Pasteurellaceae, *Fusobacterium* and *Neisseria*	**5**
	USA	15	23–77 years	Male and Female	Bronchial Flush and Brushing	↑*Prevotella*, *Streptococcus*, *Veillonella*, *Methylobacterium*, Comamonadaceae and Pasteurellaceae.	**6**
	USA	8	26-71 years	Male and Female	Oral Wash, Bronchial Flush and Brushing	↑*Prevotella*, Veillonella, *Streptococcus*, and Pasteurellaceae<br>↓*Fusobacterium* and *Neisaria*	**7**

1. Biesbroek G, Tsivtsivadze E, Sanders EA, Montijn R, Veenhoven RH, Keijser BJ, et al. Early respiratory microbiota composition determines bacterial succession patterns and respiratory health in children. Am J Respir Crit Care Med. 2014;190(11):1283-92.
2. Lemon KP, Klepac-Ceraj V, Schiffer HK, Brodie EL, Lynch SV, Kolter R. Comparative Analyses of the Bacterial Microbiota of the Human Nostril and Oropharynx. mBio 2010; 1.
3. Whelan FJ, Verschoor CP, Stearns JC, Rossi L, Luinstra K, Loeb M, et al. The loss of topography in the microbial communities of the upper respiratory tract in the elderly. Ann Am Thorac Soc. 2014;11(4):513-21.
4. Charlson ES, Bittinger K, Haas AR, Fitzgerald AS, Frank I, Yadav A, et al. Topographical continuity of bacterial populations in the healthy human respiratory tract. Am J Respir Crit Care Med. 2011;184(8):957-6.
5. Bassis CM, Erb-Downward JR, Dickson RP, Freeman CM, Schmidt TM, Young VB, et al. Analysis of the upper respiratory tract microbiotas as the source of the lung and gastric microbiotas in healthy individuals. MBio. 2015;6(2):e00037-15.
6. Dickson RP, Erb-Downward JR, Freeman CM, McCloskey L, Beck JM, Huffnagle GB, et al. Spatial variation in the healthy human lung microbiome and the adapted island model of lung biogeography. Ann Am Thorac Soc. 2015;12(6):821-30.
7. Dickson RP, Erb-Downward JR, Freeman CM, McCloskey L, Falkowski NR, Huffnagle GB, et al. Bacterial topography of the healthy human lower respiratory tract. MBio. 2017;8(1):e02287-16
