# Supplementary figures and images for: An optimized approach for processing of frozen lung and lavage samples for microbiome studies
Source: PLoS One. 2022 Apr 5;17(4):e0265891. doi: 10.1371/journal.pone.0265891 (PMC8982836; doi:10.1371/journal.pone.0265891)

A

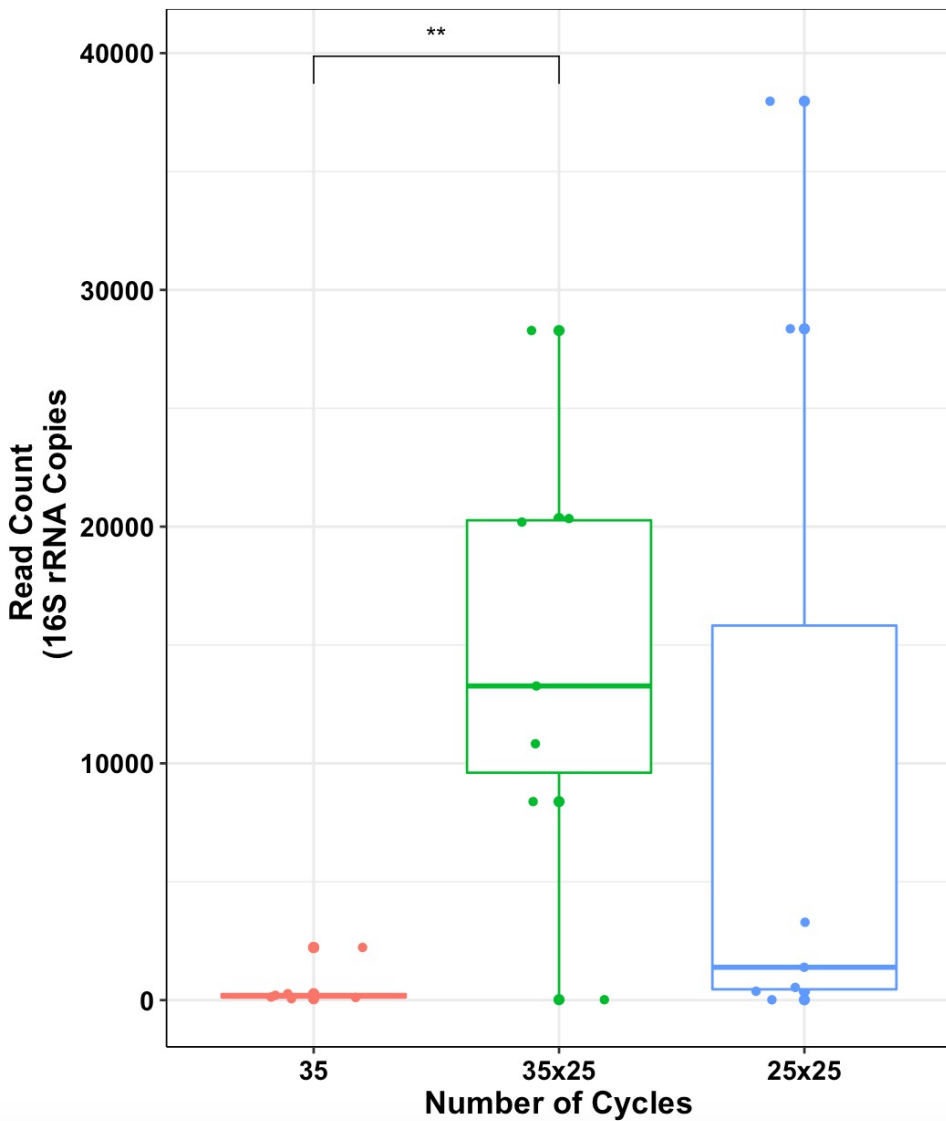

B

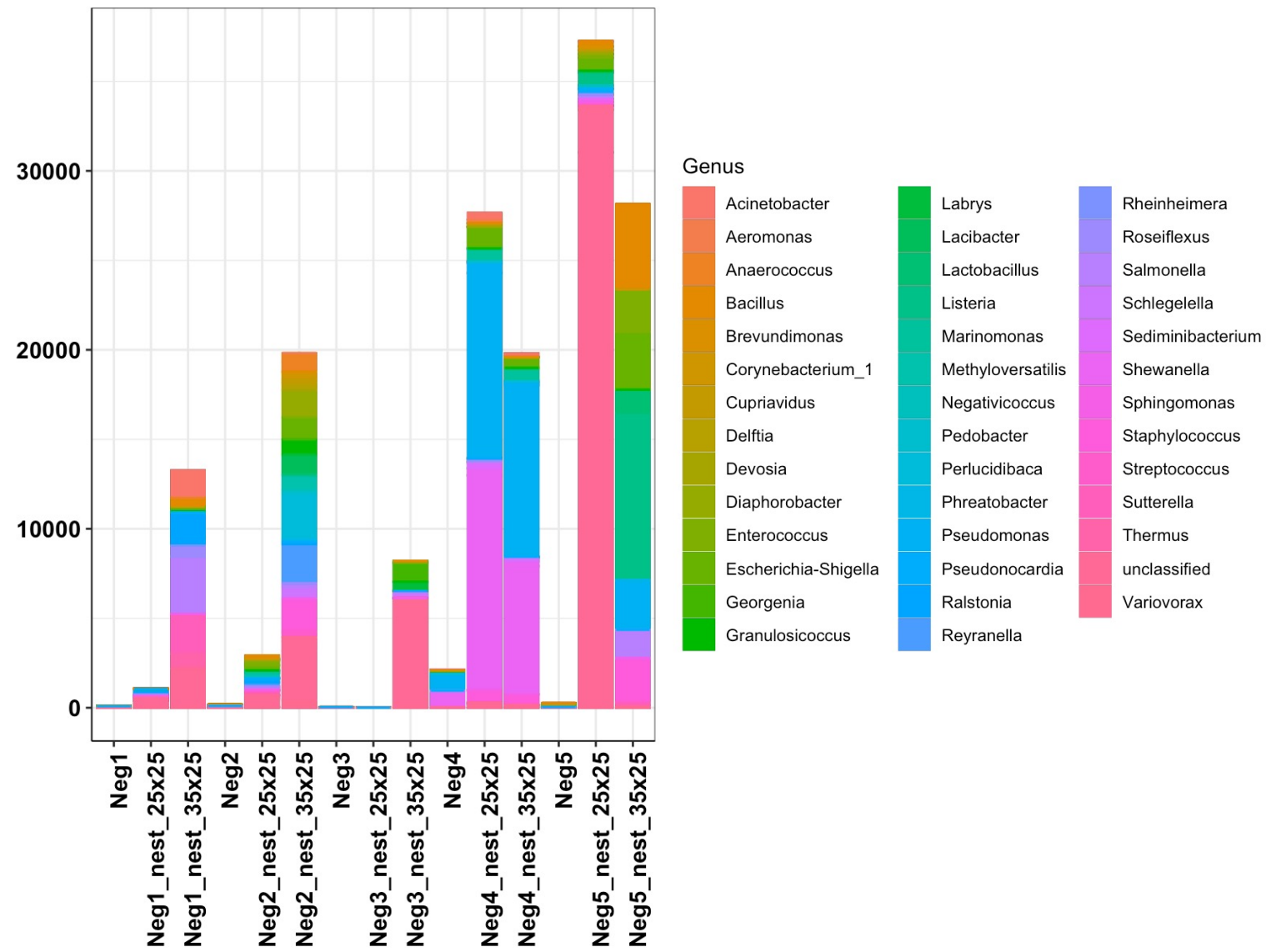

Supplement: S1 Fig — A) Data shows that nested PCR more than doubles amplification of contamination. Significant (p < 0.01) difference observed when comparing nested (35x25) and non-nested PCR. B) Nested samples exponentially increased contamination in all negative control. Thus, nested PCR samples were discarded form the analysis and from further processing. (PDF) [file pone.0265891.s002.pdf]

Read Count  
(16S rRNA Copies)

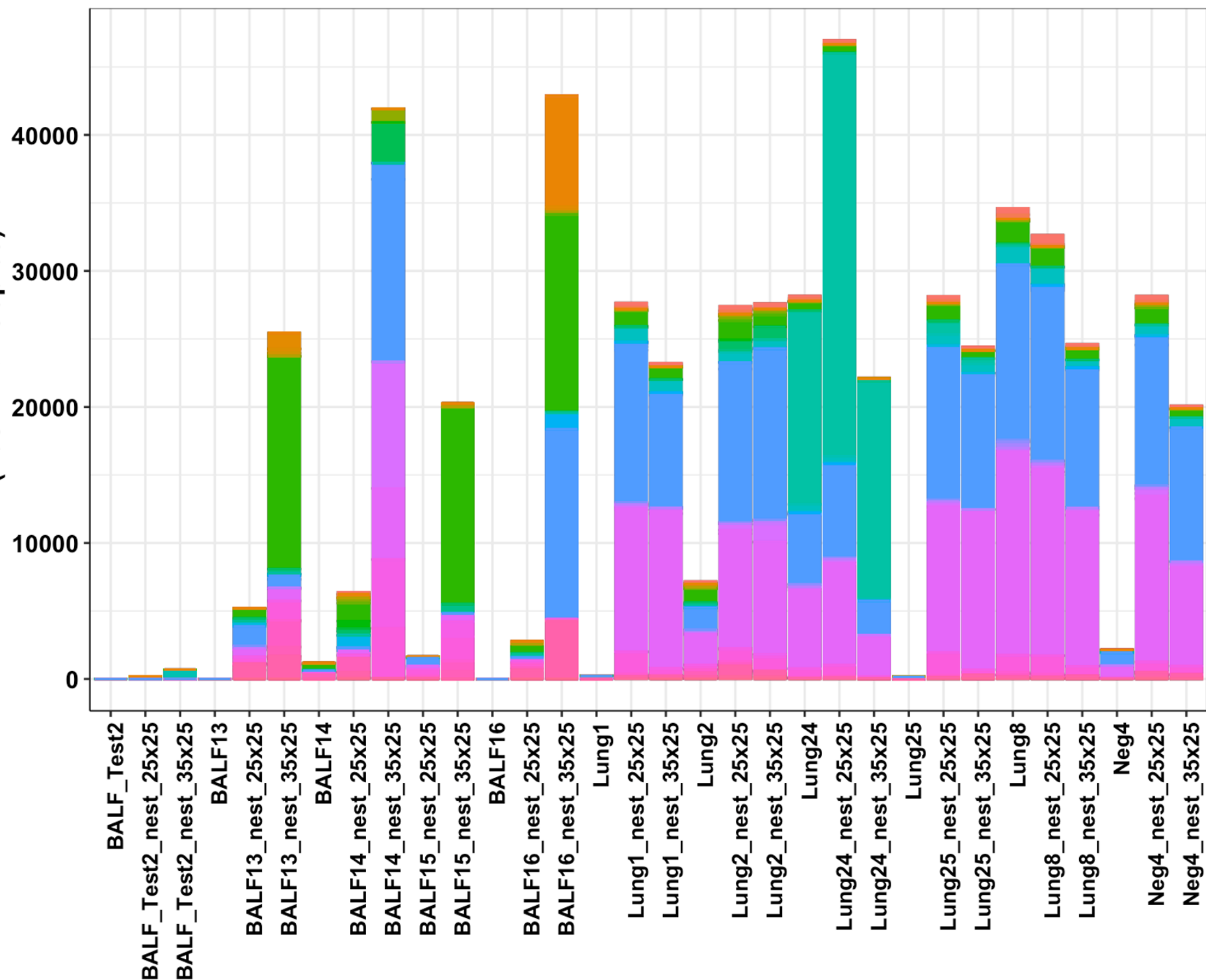

Genus

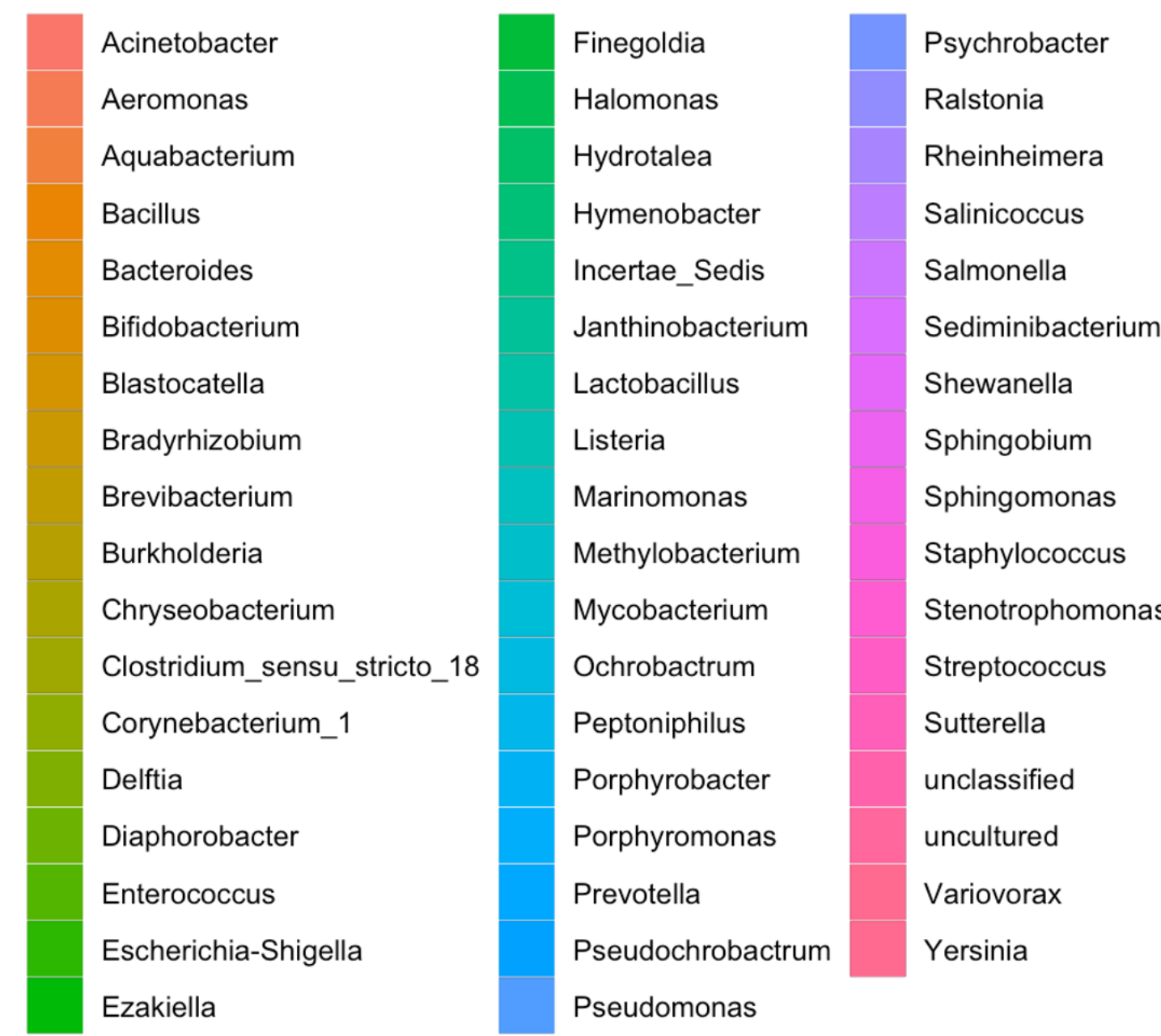

Supplement: S2 Fig — Most samples had a high level of contamination potentially from water and reagents by detecting a higher abundance of Pseudomonas and Shewanella sp. overshadowing the lung microbiome signal in most samples. (PDF) [file pone.0265891.s003.pdf]

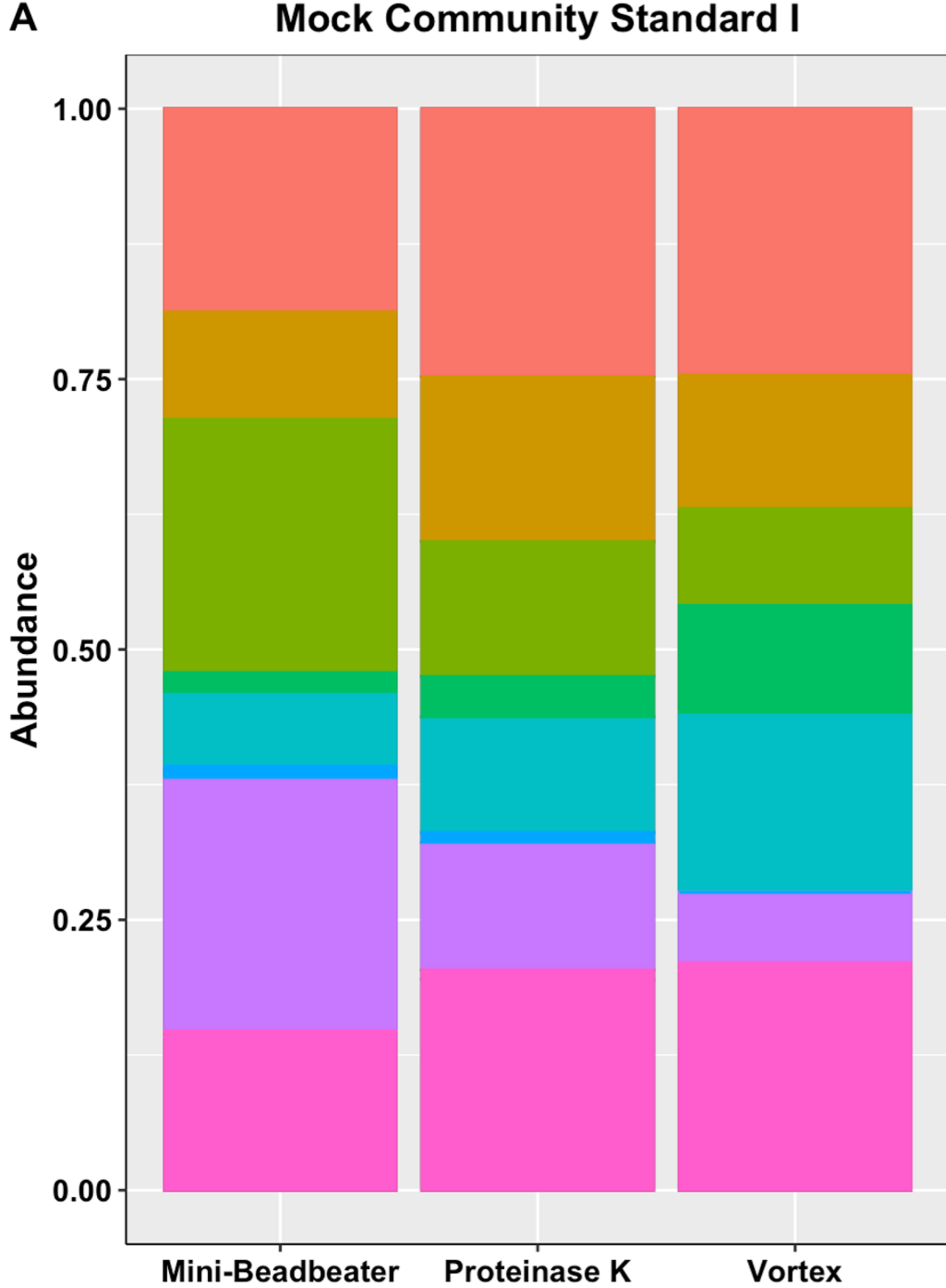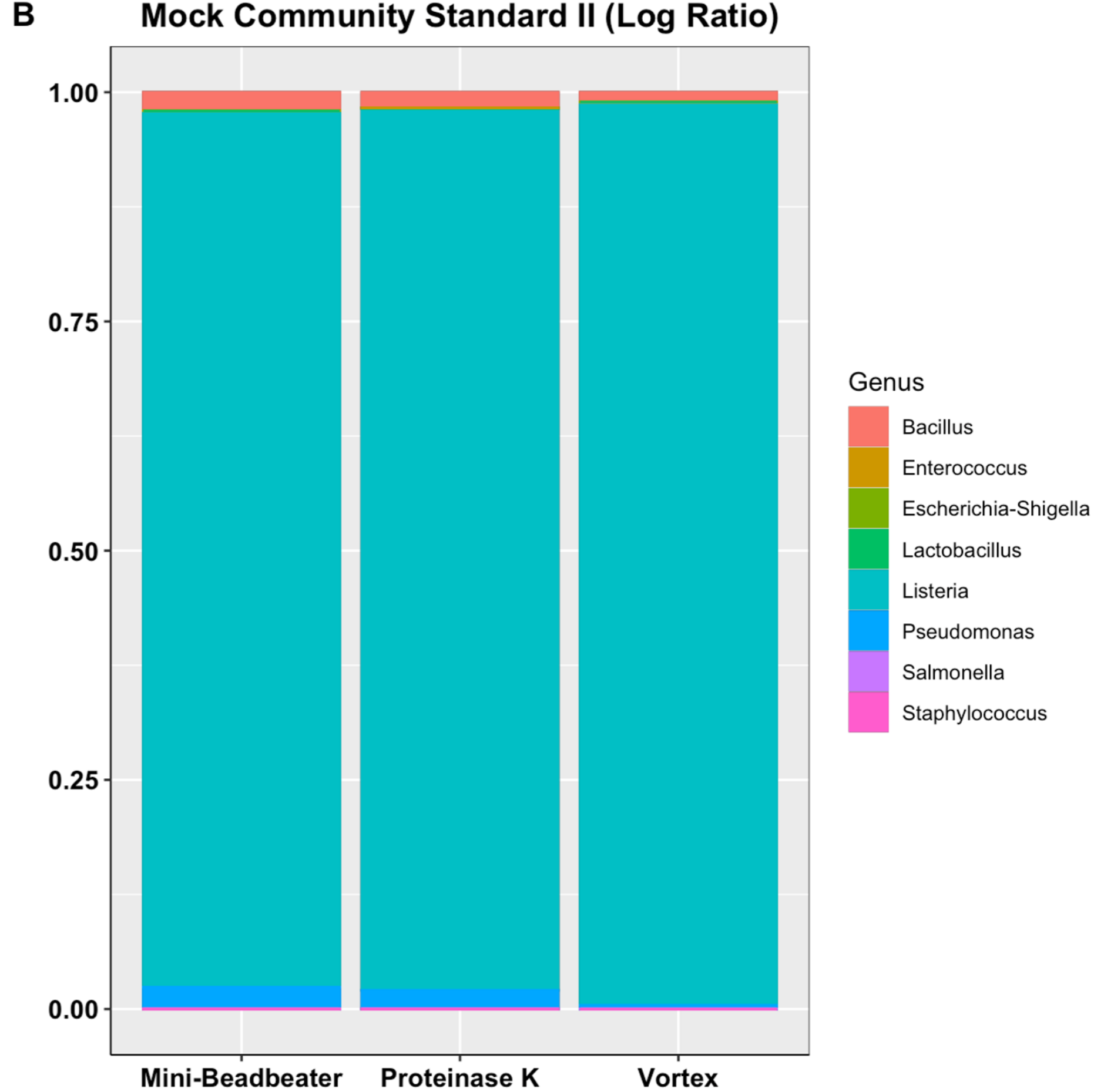

Supplement: S3 Fig — A) Mock Community Standard I (Cat# D6300) and B) Mock Community Standard II (Cat# D6310), all pre-treatment methods give a good representation of the microbial mock community as suggested by ZymoBIOMICS 16S theorical microbial composition. (PDF) [file pone.0265891.s004.pdf]

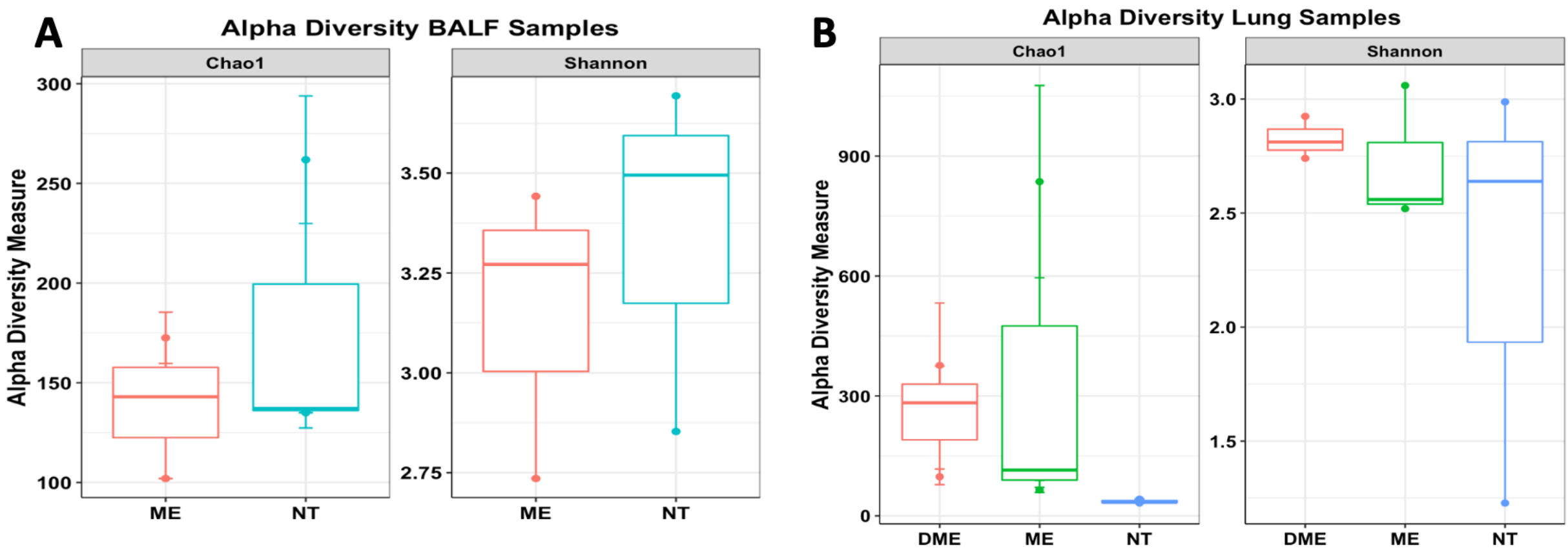

**C Bacterial Composition of NEBNext Treated and Non-treated Samples**

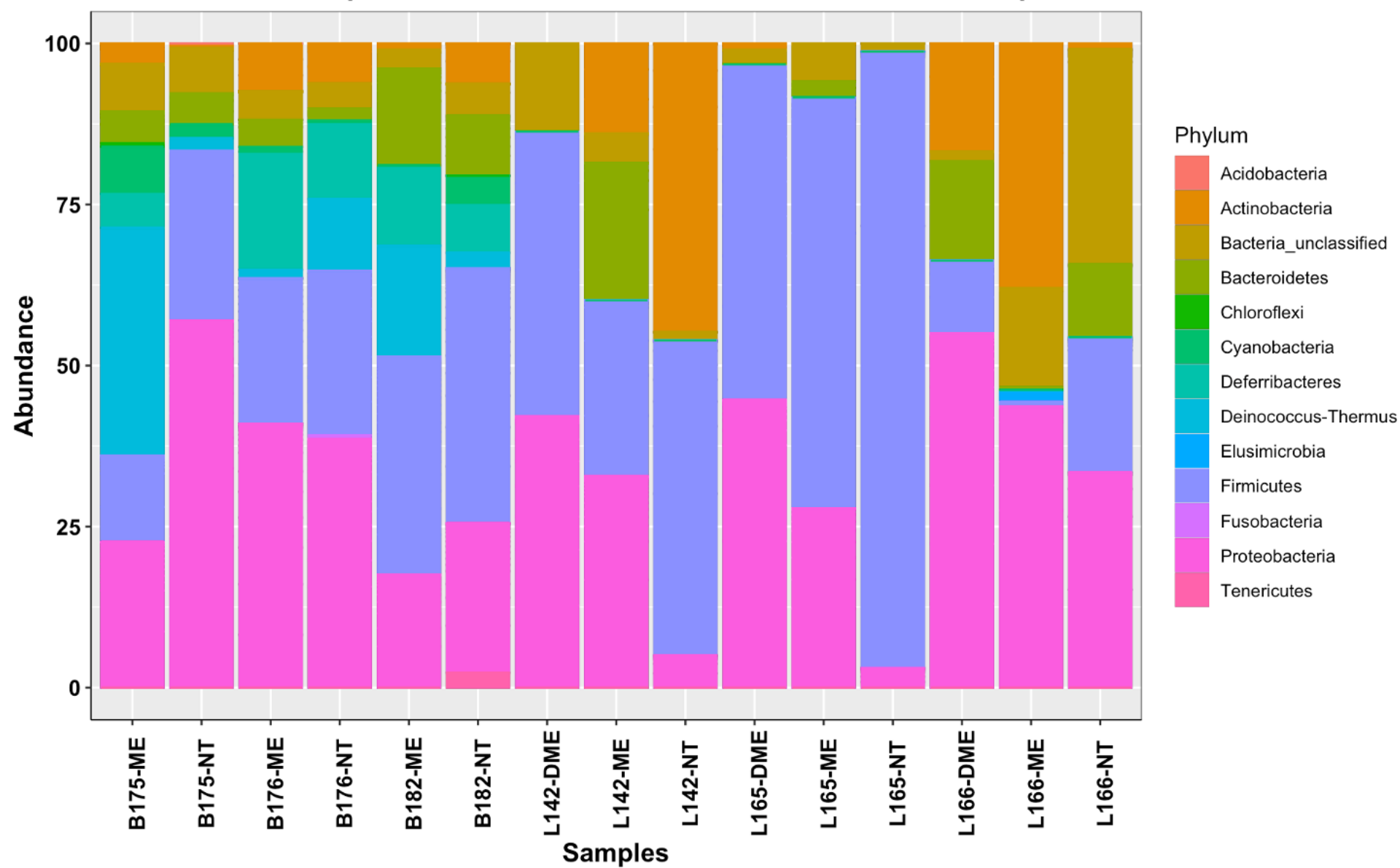

Supplement: S4 Fig — Abbreviation for non-treated (NT), microbiome enriched (ME) and diluted sample then treated with ME kit (DME). A) Alpha diversity measures shows that BALF samples, treated samples have a lower species diversity comparted to the non-treated samples. B) Alpha diversity measures shows that lung tissue samples, slight increase species richness was observed for treated lung samples. C) Bacterial composition of lung and BALF samples. Overall, samples had a higher abundance of Proteobacteria and Firmicutes bacteria. (PDF) [file pone.0265891.s005.pdf]

Primers 16S Gene GAPDH Gene

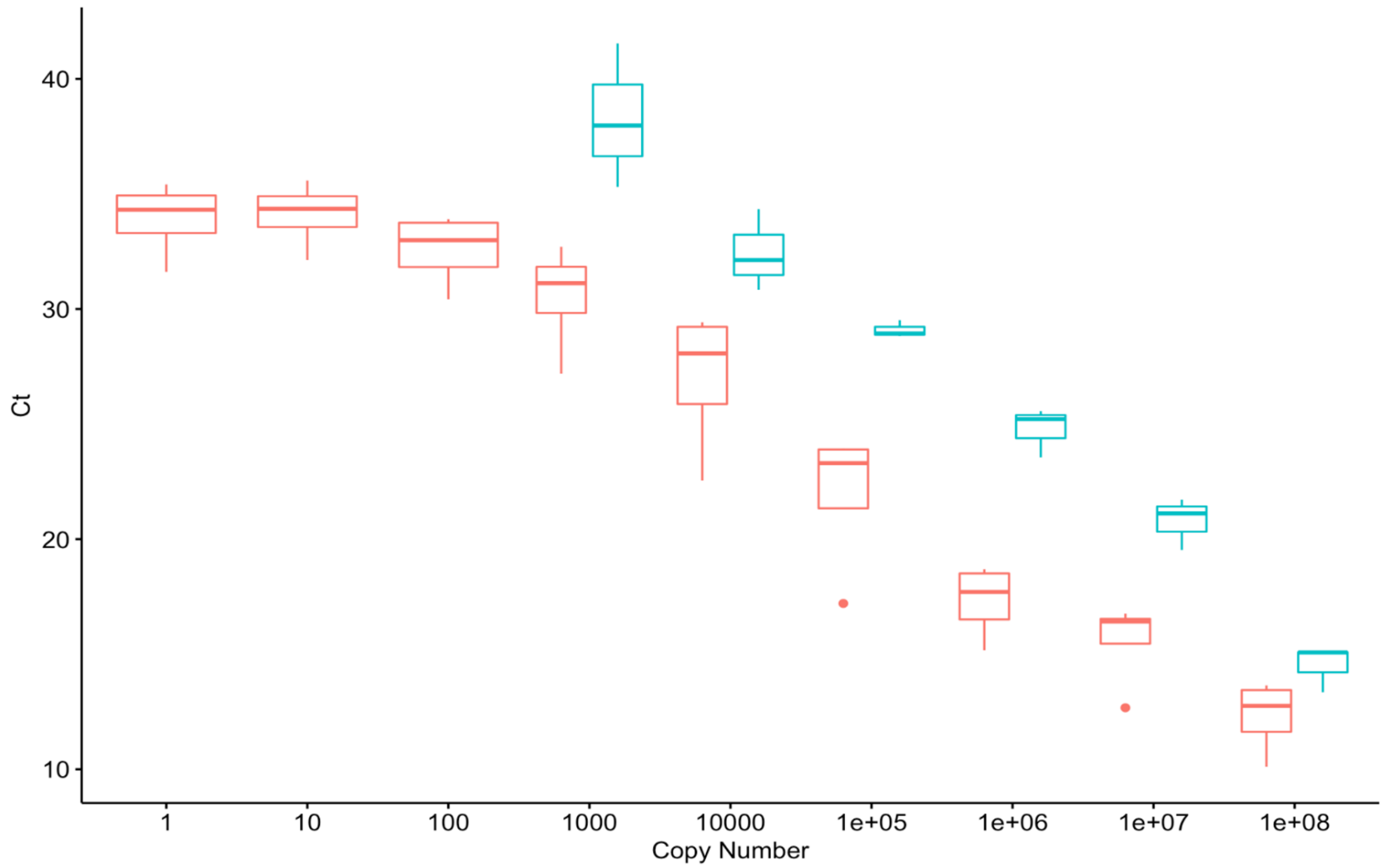

Supplement: S5 Fig — Both 16S and GAPDH genes had a low primer specificity in the qPCR assay. For 16S qPCR assay, cutoff was set to 100 copies. GAPDH cutoff was set to 1000 copies. (PDF) [file pone.0265891.s006.pdf]
